# Quality of life in pediatric oncological disease: Integrative literature review

**DOI:** 10.1080/07853890.2021.1896045

**Published:** 2021-09-28

**Authors:** Bárbara Santos, Carolina Santos, Catarina Couto, Joana Almeida, Cidália Castro

**Affiliations:** a Nurse Department, Escola Superior de Saúde Egas Moniz (ESSEM), Egas Moniz Cooperativa de Ensino Superior, Caparica, Portugal; b Centro de Investigação Interdisciplinar Egas Moniz (CiiEM), Egas Moniz Cooperativa de Ensino Superior, Caparica, Portugal; c Unidade de Investigacão e Desenvolvimento em Enfermagem (UI&DE), Monte de Caparica, Portugal; d Unidade de Investigação e Desenvolvimento em Enfermagem (UI&DE), Lisboa, Portugal

**Keywords:** Oncology, paediatric, quality of life, family, NURS

## Abstract

**Introduction:**

With the progressive technological development, that we can observe all over the world, and with the increasing rates of paediatric cancer patient survivals, there are every day more “cured” children, but those sequels whom remained will interfere in these children’s quality of life and others [1]. It’s becoming important to evaluate these paediatric cancer patients in order to evaluate the results of the treatments in their perspective, giving nursing a prestige and preponderant roll in the treatments clinical evaluation, on children’s health surveillance and monitoring, in order to increase health gains [2]. The aim of this study is to identify the paediatric cancer patients and their families needed nursing cares in order to increase their quality of life.

**Materials and methods:**

An Integrative literature review by the PI[C]OD method [3]. To answer the investigation question: “Which nursing cares should we adopt with the children and his family, with the purpose of increasing their quality of life facing a oncologic pediatric disease?”. Available articles were searched in knowledge library online (B-on), EBSCOhost database and the Scientific Electronic Library Online (SciELO). Published between 2013 to 2019. The inclusion criteria were Paediatric cancer patients and families, Nursing care and quality of life. From a total of 43 articles, 7 were included in the study.

**Results:**

There are three key thematics, namely: (1) Psycho-oncology. This one has a decisive role in the therapeutic adherence of the medical treatment, as well as in the family support [4]. (2) Family nursing. The family nurse is represented as the reference link between the Healthcare Service and the user/family, assuming the responsibility in the assurance of global health care assistance for several families, at many cases of crisis and in all the processes of health-disease [5]. (3) Needs of children and families. These children and their family have different specific needs, various and changing, that include a physical psychologic and spiritual dimension [6].

**Discussion and conclusions:**

The studies underline the importance of nursing interventions in order to improve the childs with cancer disease and their family’s quality of life. At the level of clinical practice in nursing, it is important to discuss the results of this study within the team, ensuring patient safety and the quality of nursing care based on scientific evidence.
